# Advances in Genomic Profiling and Analysis of 3D Chromatin Structure and Interaction

**DOI:** 10.3390/genes8090223

**Published:** 2017-09-08

**Authors:** Binhua Tang, Xiaolong Cheng, Yunlong Xi, Zixin Chen, Yufan Zhou, Victor X Jin

**Affiliations:** 1 Epigenetics & Function Group, School of the Internet of Things, Hohai University, Changzhou Campus, Changzhou 213022, Jiangsu, China; 15061129503@163.com (Y.X.); czx1994@126.com (Z.C.); 2 School of Public Health, Shanghai Jiao Tong University, Shanghai 200025, China; 3 Department of Molecular Medicine, University of Texas Health Science Center, San Antonio, TX 78229, USA; xiaolongcheng1120@gmail.com (X.C.); zhouy4@uthscsa.edu (Y.Z.)

**Keywords:** Hi-C, 3D chromatin, structure interaction, single cell, disease

## Abstract

Recent sequence-based profiling technologies such as high-throughput sequencing to detect fragment nucleotide sequence (Hi-C) and chromatin interaction analysis by paired-end tag sequencing (ChIA-PET) have revolutionized the field of three-dimensional (3D) chromatin architecture. It is now recognized that human genome functions as folded 3D chromatin units and looping paradigm is the basic principle of gene regulation. To better interpret the 3D data dramatically accumulating in past five years and to gain deep biological insights, huge efforts have been made in developing novel quantitative analysis methods. However, the full understanding of genome regulation requires thorough knowledge in both genomic technologies and their related data analyses. We summarize the recent advances in genomic technologies in identifying the 3D chromatin structure and interaction, and illustrate the quantitative analysis methods to infer functional domains and chromatin interactions, and further elucidate the emerging single-cell Hi-C technique and its computational analysis, and finally discuss the future directions such as advances of 3D chromatin techniques in diseases.

## 1. Introduction

Although the diverse cell types of an organism share the same DNA information, their genomes undergo quite different structural and organizational changes during differentiation and replication; and such changes affect gene expression and cellular functions via different information routes.

In vivo, human genome functions as a folded three-dimensional (3D) chromatin polymer. Nearly half a century ago, gene position on a single chromosome was considered a main determinant of gene activity due to the lack of an advanced profiling technique. The regulatory activities were simplified into a genetic one-dimensional sequence underlying the expression level [1]. The 3D physical location of regulatory elements and corresponding targeted genes have gained prominence with the most recent sequence-based profiling technologies such as high-throughput sequencing to detect fragment nucleotide sequence (Hi-C) and chromatin interaction analysis by paired-end tag sequencing (ChIA-PET) [2,3]. Thus, more functionally diversified regulatory elements (REs), including enhancers, silencers, insulators, and boundaries, have been identified to act collaboratively with active promoters via long range tethering or chromatin looping mechanisms [4]. The looping paradigm has now been recognized as a basic principle of gene regulation.

Through genome-wide assessing of chromatin interaction and characterizing the global contacts between the regulatory elements and targets, the spatial architecture of the genome is found to be indispensable functional units in the nearly all of the transcription and translation processes [5,6,7,8]. The functional units as promoters, enhancers, silencers, and insulators can regulate their respective targets in both in cis and in trans manners under spatiotemporal chromatin conformations. For example, Homeotic complex D (HoxD), with up to 13 homeobox gene paralogs following collinearity in organization and expression, is regulated with the sequential chromatin opening and promoter-enhancer looping. The entire HoxD complex keeps in one topologically association domains (TAD) when undifferentiated, but segments into two TADs during differentiation, together with the locus transition between these two TADs, related to cell-type identity [9].

Furthermore, huge efforts have been made in developing novel analysis methods to better interpret the data and gain biological insights. However, the full understanding of genome regulation requires a thorough knowledge in both genomic technologies and their related data analyses.

In this work, we summarize the recent advances in genomic technologies in identifying 3D chromatin structure and interaction, and illustrate the quantitative analysis methods on processing data and inferring functional domains or chromatin interactions, and further elucidate the newly emerging single-cell Hi-C technique and its computational analysis, and finally discuss the future directions such as advances of 3D chromatin profiling technique in diseases.

## 2. Nuclear Organization and Functional Elements

During cell differentiation and transcription, the human genome functions as a folded 3D chromatin structure. These processes cover the large-scale folding of whole chromosomes or smaller genomic regions, and the reorganization of local interactions between enhancers and promoters, mediated by the binding of transcription factors and chromatin looping. The higher-order organization of chromatin is also influenced by the specificity of the contacts that it makes with nuclear structures such as the lamina [8].

Within condensed chromatin compartments, there exist functionally genetic elements called TAD, serving as a pervasive structural feature in the genome organization. Moreover, the occurring topological domains keep stable across the diverse cell types, and remains highly conserved by cross-species studies, indicating that TADs are inherent and important functional units in mammalian genomes [10,11,12].

Thus, to uncover how the human genome is spatially organized and condensed within the cell nucleus is a prerequisite to understanding genetic regulation mechanisms in normal differentiation, development, and even dysregulation in disease.

### 2.1. The Structure and Functional Units of Chromosome

Diverse contact structures and models have been raised so far, such as the early proposed active chromatin hub (ACH) related to hypersensitive genomic sites [13]. ACH is formed by direct contact between enhancers and promoters of targets by sequence looping [14]; and, ACH-type contacts were reported in the mouse gene CD8 locus [15], and in human AML1/RUNX1 [16] and CFTR loci [17].

TAD is a discretely folded structural entity with specific chromatin activities, normally it takes the size of kilo to mega base pairs. The central regions in TADs have highly frequent chromatin interactions, supporting the viewpoint that TADs dictate tissue-specific genes’ and enhancers’ activities [18,19,20]. In addition, there exist clustered housekeeping genes enriched within TAD boundaries, together with high concentrations of CTCF and cohesion binding events; and, the inter-interactions between TADs are often inhibited by CTCF binding [8].

Within nucleus, the inner envelope is covered by lamins and other types of proteins, both of which form the nucleus laminas. Nucleus laminas have the key leverage on the spatial organization of chromosomes through contacts with chromatins while sustaining spatial positions on the inner nucleus. Lamina-associated domain (LAD) is a recently identified structural unit associated with condensed chromatin, often bound by nucleus lamina. LADs have a median size of ≈0.5 Mb, with a relatively low gene density where most of these genes are transcriptionally inactive, suggesting that the nuclear lamina has a repressive role in gene regulation. LADs also participate in the two main roles during cell differentiation when constitutive LADs (cLAD) keep attached to laminas, and facultative LADs (fLAD) become disaffiliated from laminas due to the corresponding activated genes. After mitosis, some LADs can return to the nucleolus periphery, which was reported as a sequence specificity although the underlying mechanisms remain unclear [21,22].

Noticeably, within TADs, there exists two highly dynamic architectures of submegabase scale: (I) smaller-size domains, named sub-TADs [23,24,25], and (II) chromatin loops [24,26], which are closely related to cell differentiation and development.

### 2.2. High-Throughput Profiling 3D Techniques

Deciphering the mechanisms controlling chromosome folding and interpreting their roles in gene regulation become the focus in the current epigenetic study. Recent high-resolution microscopy and conformation capture-based techniques have revealed that chromatin has a complicated structure, varying across different organisms and cell types.

**Figure 1:**
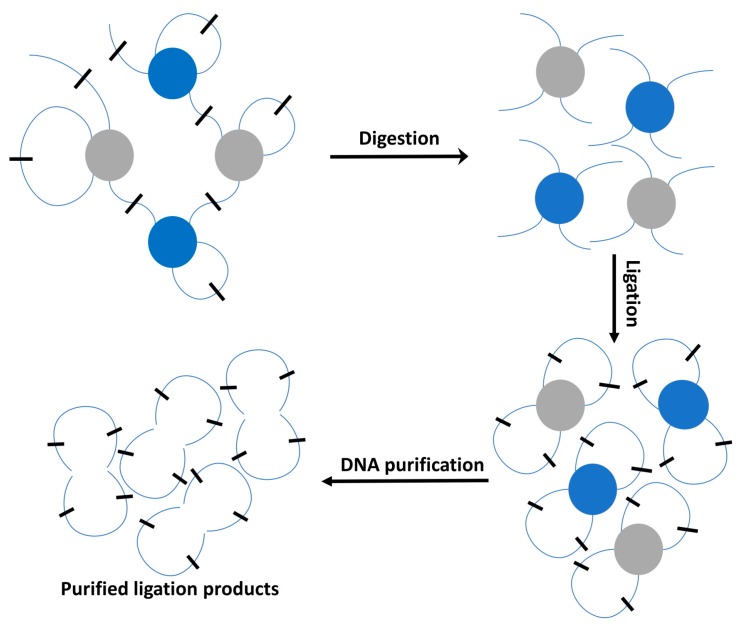
Schematic illustration of the experiment pipeline for the Chromosome, Conformation and Capture (3C)-based method and its derivatives. Basically, the 3C capture technique includes digestion, ligation, DNA purification, and the following sequencing for the resulting ligation products.

Until now, sophisticated profiling platforms in mapping chromatin contacts generate high-throughput sequencing data with deep insights into the 3D formation of chromatin interactions, and into their roles in the chromatin organization and cellular function [3,10,27]. The Chromosome Conformation Capture (3C)-based methods are major genomic technologies widely used for determining chromatin structure and interaction (Figure 1). Typically, after chromatin fragmentation being digested by restriction enzyme, being chemically cross-linked, digested, end-repaired, and proximity-ligated with a biotinylated bridge adaptor, and the resulting ligations are further sheared, affinity-purified by streptavidin bead immobilization, and subjected to traditional PCR amplification-based ligation capture or next-generation library preparation for paired-end sequencing [28]. Ligated products, which are considered as putative chromatin contacts, are further subjected to the quantitative analysis for determining their genomic distances and locations with different resolution levels.

PCR amplification-based ligation capture techniques that are mainly composed of the typical 3C-based technique and its extensions, such as Chromosome Conformation Capture on Chip (4C) [29,30], and Carbon-Copy Chromosome Conformation Capture (5C) [31]. The sequencing-based techniques generally cover multiplexed 3C sequencing (3C-seq) [32], 4C combined with high-throughput sequencing (4C-seq) [33], capture-C [34], Hi-C [3], Tethered Conformation Capture (TCC) [35], and Targeted Chromatin Capture (T2C) [36]. There are also other techniques in the field, but due to the space limit, we just name a few representative platforms as listed above.

## 3. Advances in Statistical and Computational Analyses

### 3.1. General Pipeline for Preprocessing High-Throughput Profiling 3D Data

**Figure 2:**
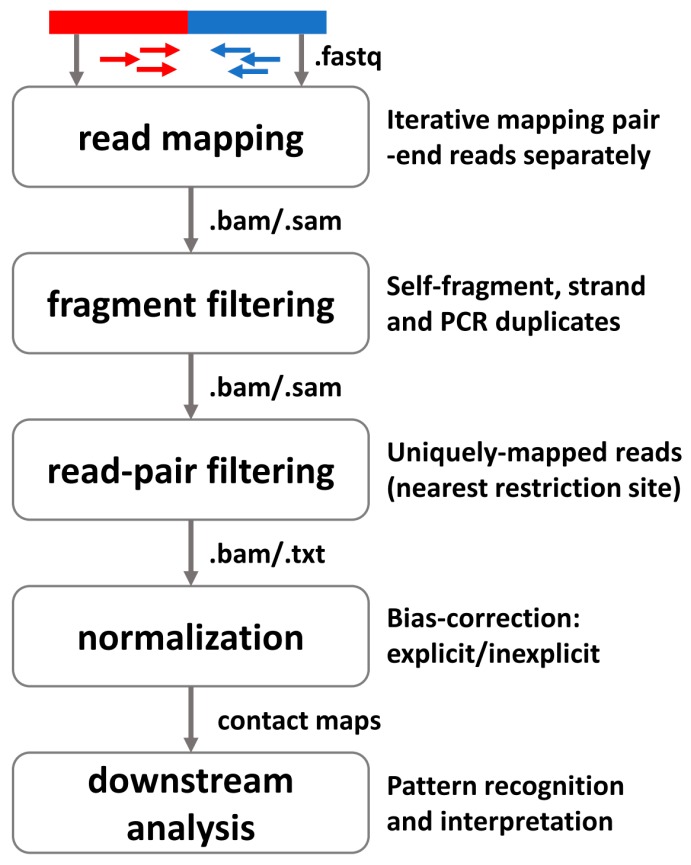
Schematic illustration of the computational analysis pipeline for the 3C and its derivatives, particularly the Hi-C technique. In analysis sequence, the pipeline contains raw reads mapping, fragment filtering, read-pair filtering, normalization and downstream analyses of contact maps.

Given the diverse experimental protocols, there requires specific consideration in designing the computational algorithms and pipelines for characterizing 3D chromatin interaction. In this section, we illustrate the basic principle and general pipeline for preprocessing high-throughput profiling 3D data. A typical sequence-based 3D data preprocess procedure includes raw reads mapping, fragment filtering, read-pair filtering, normalization, and downstream analysis (Figure 2). For the initial mapping procedure, because the end distances in the ligation products are of various lengths, from a few base pairs to kilobases, thus it is necessary to iteratively map each pair-end reads in processing the raw sequence reads.

In short, Figure 2 lists the other necessary analysis procedures as fragment and read-pair filtering, before the bias-correction normalization. Then downstream analyses focus on pattern recognition and deep interpretation of the spatial interaction among chromatin structures.

### 3.2. Progresses in Modeling and Analyse of 3D Chromatin Interactions

In addition to noticeable advancements in profiling techniques, statistical models and quantitative analyses are a prerequisite in discovering the chromosome folding mechanism, together with its origin and function in diverse cellular activities. In this section, we review recent progresses in chromosome modeling with a focus on computational analysis of identifying chromatin interactions.

The Strings and Binders Switch (SBS) model was introduced based on the hypothesis that the chromatin conformation occurs during the interaction of specific binding sites with diffusing binding factors. SBS model has shown that it can recapitulate scaling and dynamic folding properties of chromosome organization in the spatial and temporal dimensions [37,38].

To reveal the causal underpinnings of complex diseases, there is still an open challenge to distinguish the gene targets of a distal regulatory element from other adjacent coding genes. Whalen et al. presented a computational method, TargetFinder, to reconstruct the regulatory landscapes from multiple genome-wide features [39]. It was claimed that the resulting models could accurately predicate the individual enhancer–promoter interactions across multiple cell lines with a much smaller false discovery rate than using the closest gene. The authors further evaluated the genomic signatures contributing to the claimed accuracy, and revealed combined interactions among structural proteins, transcription factors, epigenetic modifications, and transcription that together distinguish interacting from other enhancer-promoter pairs. In summary, the method was reported to accurately predict the interactions up to 2 Mb apart at a high resolution and identify minimal sets of predictive features quantified by genomic region; together with a focus on high-resolution intra- rather than inter-TAD interactions [40].

Because 3D genome structures are highly plastic and diverse among cells even in an isogenic sample, it is still a major task to inferring structure-function linkages. Recently, based on ensemble-averaged and single-cell Hi-C data, Dai and their colleagues reported an approach to comprehensively identify 3D chromatin clusters occurring frequently across a population of genome structures [41]. At the macrodomain resolution on lymphoblastoid cells, they identified an atlas of stable inter-chromosomal chromatin clusters, defined as Regulatory Communities. They further showed centromere clustering and transcription factor (TF) binding could significantly stabilize the communities that were found to be cell specific. This indicates that the connection between expression variability and genome structure [40]. Lan et al. proposed a Mixture Poisson Regression Model and a power-law decay background to define a highly specific set of interacting genomic loci and regions [15]. By integrating with multiple ENCODE Consortium resources with the Hi-C data, DNase-seq data and ChIP-seq data for 45 TFs and 9 histone modifications, they classified 12 different clusters of interacting loci with two distinguished types of chromatin linkages. They further found that cluster 9 was highly enriched for three TFs (GATA1, GATA2 and c-Jun) and three chromatin modifiers (BRG1, INI1, and SIRT6). Their work provides genome-wide evidence that the Hi-C data identify sets of biologically relevant interacting loci [14].

To assess the chromatin domains and their positional association, Molitor et al. introduced multiscale correlation evaluation (MCORE), based on the fluctuation spectrum of mapped sequencing reads to quantify and compare chromatin patterns with diverse scales. Through integrating multiple sources from chromatin immunoprecipitation, RNA expression, DNA methylation, and 3C experiments, the approach was claimed capable of revealing the positional relationships on different genomic scales [41].

Despite plenty of cis-regulatory sequences annotated, it is still challenging to identify their target genes in the human genome. Previous strategy is to profile the long-range looping interactions for those elements with 3C-based techniques, but they lack either resolution or coverage depth satisfying the whole-genome and precise capture of chromatin interactions.

Ren and his colleagues reported a comprehensive chromatin interaction map at 5–10 kb resolution for human fibroblasts using a genome-wide Hi-C sequencing technique [28]. Through determining over one million long-range chromatin interactions, they concluded the general principles of chromatin organization at different types of genomic features, together with the dynamics of promoter-enhancer contacts after tumor necrosis factor-α (TNF-α) signaling in these cells. Long-range interactions within transcription regulatory elements implement key roles in gene activation, epigenetic silencing, and chromatin organization. They further claimed that the established 3D chromatin landscape for a particular cell type is comparatively stable and could function cell-specifically on the selection or activation of target genes [27].

Genetic transcription activities depend on tissue-specific chromatin architecture and flexible localization among the involved transcription and regulation elements. The inherent organization and coordination mechanisms are still under investigation. Bortle et al. explored recent findings, and focused on highly conserved multiprotein complexes composed of insulator and Polycomb group proteins, which were identified with functions in interceding long-range interactions and nuclear organization [42]. Furthermore, chromatin contacts for inter- and intra-promoters and other elements present cell-specific epigenomic characteristics. Network analysis is another way in modeling the chromatin interactions. Recently, a chromatin assortativity-based method was proposed to combine the epigenomic landscape of a specific cell type with its chromatin interaction network. By high-resolution promoter capture Hi-C, Hi-Cap, and ChIA-PET data from mouse embryonic stem cells, the authors studied promoter-centered chromatin interaction networks, and further quantified the presence of specific epigenomic features in the chromatin fragments for the network nodes. It was reported the method could identify the proteins or chromatin marks mediating the genomic contacts [43].

### 3.3. Processes in Inferring 3D Spatial Structure

**Figure 3:**
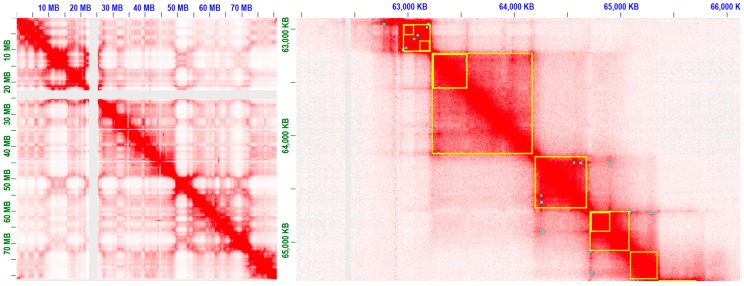
Extract contact map from human B-lymphoblastoid Hi-C data (Rao et al. [24]) and focus on individual chromosome (chr17). Contact domains and peaks are highlighted in yellow boxes and cyan points, respectively. Peak detection is used to distinguish between functional contacts and contacts that are due to random polymer looping or other confounding factors.

Both the well-established light microscopy-based cell-imaging and most recent molecular 3C-based techniques provide researchers with the unprecedentedly precise insight into human genomics. The human genome exists as a stereoscopic entity within the nucleus, and lineage-specific transcriptional activities related to cell identity and fate are performed under the 3D context. Molecular 3C-based techniques have many unique advantages in 3D chromosome conformation studies, which make it possible to identify the conformations between cells in the population. Hi-C contact maps are the foundation for Hi-C data analyses of 3D spatial structure, as depicted in Figure 3. High quality Hi-C contact map is an important prerequisite for 3D chromosome conformation study, and several typical tools for Hi-C data processing are listed in Table 1.

To correct the copy number bias in the Hi-C interaction matrix, Wu et al. recently proposed a linear regression-based chromosome-level adjustment method called caICB [45], which is based on the ICB protocol, to correct for the bias. They proposed a chromosome-adjusted iterative correction method that significantly improved in terms of eliminating copy number bias, when compared to the original iterative correction [44].

TAD is a discretely folded domain with self-interacting chromatin in its central region, and normally such a structural entity ranges from kilo- to mega-basepairs. Since the current 3C-based sequencing data is inevitably contaminated with systematic biases, the ICE (iterative correction and eigenvector decomposition) technique was proposed to identify the local chromatin states, global chromosomal interactions, and the conserved chromatin organization [45]. To infer the hierarchy of the nested structure, TADtree was recently introduced based on empirical distributions of contact frequencies within TADs [54]. TopDom is another easy-to-implement pipeline to study cross-tissue TAD conservation [55]. To tackle terabase-size data for those with less informatics experience, Juicer is recommended as a one-click system for Hi-C experiments, although essentially it needs much more parallel computing resources [56].

Proper gene expression requires communication with the corresponding regulatory elements scattered across the chromosome. The physics of chromatin fibers imposes a range of constraints on such communication. The molecular and biophysical mechanisms for chromosomal communication are key issues in the spatial organization of chromosomes. Dekker et al. proposed a topological machine with the claimed function of setting up and exploiting a 3D genome organization to both promote and censor intra- and inter-chromosome communication [57]. Through the overview of 3D genome organization principles in mammalian cells, Gorkin et al. studied the emerging relationship between genome organization and lineage-specific transcriptional regulation, and argued their inextricable linkages with cell pluripotency [58].

To infer 3D spatial associations within TADs from histone modification, chromatin accessibility and RNA-seq profiling resources, EpiTensor was proposed to computationally identify sets of hotspots as key elements stabilizing the 3D interaction. Through further study on diverse cell types, the identified hotspots were claimed to complete with higher chromatin, transcriptional activity, and enriched TF and ncRNA binding [59].

Besides considerable genome-wide study on chromosomal architecture by 3C and Hi-C techniques, the recent in situ DNase Hi-C was reported to demonstrate the inactive murine X chromosome adopts a bipartite structure. The in situ DNase Hi-C relies on the endonuclease DNase I, rather than on a restriction enzyme to digest chromatin as traditional Hi-C does. Furthermore, through comparison with traditional Hi-C libraries, it is claimed that in situ DNase Hi-C has a higher effective resolution. The advantage brings forward much more opportunities in higher sequencing depth or hybrid capture techniques [28].

## 4. Advances in Single Cell Hi-C Computational Analyses

### 4.1. Computational Methods to Infer Chromatin 3D Structure from Single-Cell Hi-C Data

**Figure 4:**
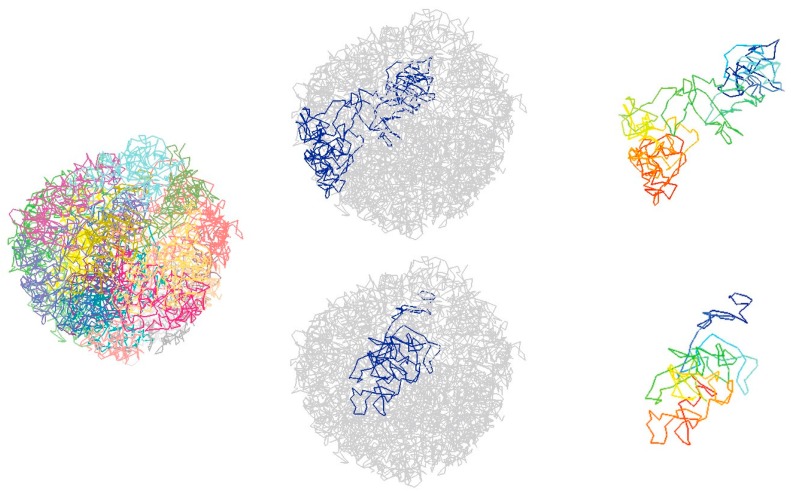
Three-dimensional (3D) structure of haploid mouse embryonic stem cells (mESCs) with the chromosomes colored differently and two chromosomes are shown separately Single-cell Hi-C data comes from Stevens et al. [66].

Owing to the great improvement in the Hi-C protocol [60], single-cell contact maps can be extracted from Hi-C data through following several steps, including the trimming of reads, mapping the reads to the reference genome, and the filtering of the mapped reads and read pairs at several different levels [61]. From each extracted single cell Hi-C contact map, chromatin 3D structures can be then inferred by using computational methods, as depicted in Figure 4. Two main types of computational methods are discussed in this section, consensus methods and deconvolution methods.

Consensus methods transform the frequency of read pairs into pairwise distances and store the distances in a matrix, in which each element can be visualized as a point in 3D space. Interaction frequencies, nuclear envelope, nuclear pore complexes, and nucleoli have been used as constraints to the reconstruction of the 3D structures. However, each distance is based on an average of multiple structures in that population and is not suitable for the triangle inequality principle. As a result, the structure inferred from the average of millions of cells will differ from structures derived from the subpopulations of cells and will typically not represent any of the structures in individual cells [62,63]. Semi-definite programming techniques have been applied to find the best consensus structure fitting the observed Hi-C data and a golden section search has been used to estimate the correct parameter for converting the contact frequency to spatial distance [64], which makes it possible to recover the correct structure in the noise-free case. Bayesian statistical models have also been used to study the consensus structures and structural variations of chromatins from the Hi-C data. The systematic biases and account for observational noise sources can be removed properly by rigorous statistical inference, and sequencing depth variations can be explicitly modeled by Poisson distributions [65].

Consensus methods are usually used to identify unique 3D structures and assess chromosomal structural heterogeneity, but cannot fully capture the 3D structural heterogeneity within a cell population [62]. They provide a possible approach to explore single cell 3D structure.

To identify structurally plausible, unobserved substructures, deconvolution methods are applied to seek an ensemble of 3D structural solutions and perform well in capture the inherent heterogeneity of chromosome structures in a cell population. There are two main deconvolution methods, namely structural deconvolution and matrix deconvolution. Structural deconvolution is applied at the 3D structure reconstruction level, and the resulting structural ensemble can be clustered to study the underlying structural variability and sub-population constituents. Matrix deconvolution is applied directly on contact frequency matrices and are usually faster than structural deconvolution, but the substructures recovered might not be physically plausible. Implementing solid-phase ligation is applied to improve the signal-to-noise ratio and enable a detailed analysis of the inter-chromosomal interactions [35]. TADs are used to search for a set of contact frequency matrices that optimally reflect the proportions of each predicted substructure in the cell population [67]. Combinatorial cellular indexing is applied to separate the cells by karyotypic and cell-cycle state differences, and to identify cell-to-cell heterogeneity in chromosomal conformation [68].

Deconvolution methods enable the inference of the main substructures that exist but require extensive computational resources. It is also not quite sure how accurate the deconvolution methods could be and whether the current data allows for the well-determined estimation of structural subpopulations.

### 4.2. Challenges for Single Cell Hi-C Computational Analyses

**Figure 5:**
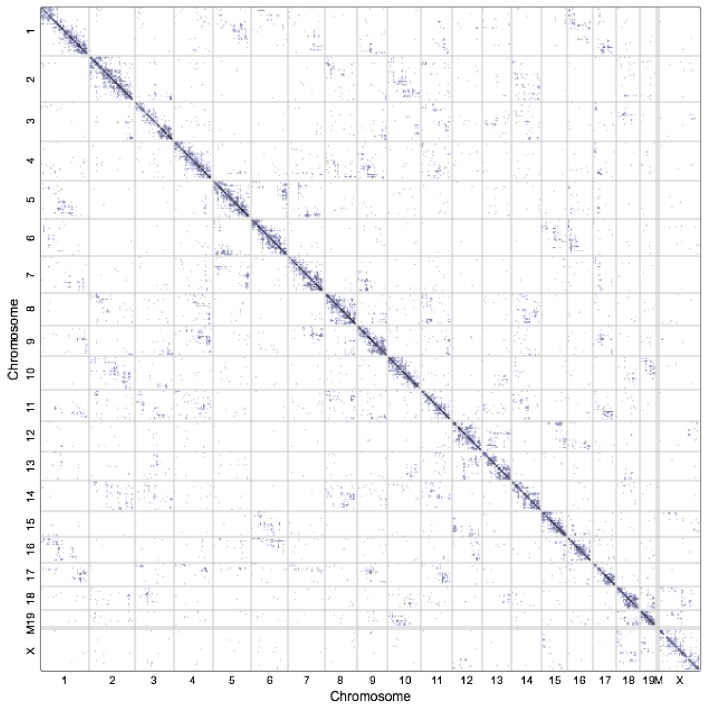
Single-cell Hi-C contact map of haploid mESCs, sparsity of the values in the distance matrix is the significant weakness Single-cell Hi-C data comes from Stevens et al. [66].

A crucial issue with single-cell Hi-C chromosome contact information is the inherent sparsity of the contact frequency maps [69,70], as depicted in Figure 5.

The genome coverage from the richest data sets was as low as approximately 2.5%, and the sparsity of genome coverage even led to concerns over the success rate of the single cell Hi-C protocol [60]. One approach to alleviate the sparsity of single-cell Hi-C data is to computationally generate the missing distances by referring to the observed contacts, but additional noise may be introduced at the same time, which dominates over the more accurate local distances. By combining the shortest-path derived distances with appropriate weights to reduce the influence of noise, the Manifold based optimization approach is a good choice to reconstruct 3D structures consistent with the chromosome contact maps [71]. Deep sequencing of single-cell Hi-C ligation products may be a solution to enable the reconstruction of 3D chromatin conformations with high confidence.

Another major inherent limitation for single-cell Hi-C experiments is the technical noise. Quality control of sequencing data is crucial to avoid technical artifacts. For single-cell sequencing, technical noise is mainly due to low amounts of starting material, often resulting in variable capture efficiencies [72]. Sequencing of negative controls is applied to reduce the reagent contamination and sample cross-contamination. Mapping efficiency or coverage cut-offs can be used to eliminate cells that have performed much worse than the average.

Additionally, the two copies of human autosomal chromosomes are difficult to be distinguished in single-cell Hi-C experiments, which can complicate the 3D structure reconstruction. The genomic distribution of the digestion sites of the restriction enzyme used can also be an important limitation to the Hi-C map resolution.

## 5. Conclusions and Future Perspectives

Despite that we have witnessed a booming field of 3D genome organization studies within a short period, we should be cautious that some important caveats have been noted about the nature of the contacts detected by ligation-based methods, especially when interpreting results obtained with these techniques [8]. It is necessary to integrate multi-source information and multi-level approaches in analyzing transcriptional regulation and their functional characteristics [73].

In addition, many issues depend on the improvement of genomic techniques and the development of sophisticated statistical and computational methods. We want to point out the devoid of substantial applications of 3D chromatin structure and interaction in disease biology.

### 5.1 Advances in 3D Chromatin Structure Interaction in Diseases

With the development of 3D genome technology, many labs have promptly applied 3C-based high-throughput profiling techniques for disease research. Here, we review a few representative studies to demonstrate aberrant 3D chromatin structures and interactions in human diseases.

Hi-C contact frequencies have been found to be tissue-specific and to influence the translocation partner selection in human diseases [74]. Chromosomal 3D structure has been indicated to have an intricate relationship with gene expression in breast cancer [75] and leukemia [14]. Aberrantly amplified distant estrogen response elements reduce transcription of the proximal target genes in luminal breast cancer [76].

Noticeably, the recent 3C-based technique revealed that genomic duplications in human patient cells and mice can lead to the formation of new chromatin domains (neo-TADs), and such a process determines their corresponding pathology outcomes [77]. Enhancer hijacking is recently proposed in the rearrangement of TAD boundaries, which mediates cancer-related gene overexpression in colorectal cancer [78]. Genetic mutations can disrupt chromosome neighborhood boundaries and then activate oncogene in many types of cancer [79].

Due to hypermethylation at cohesin and CTCF binding sites human *IDH* mutant glioma have dysfunction of chromosomal topological domains and allows oncogene expression [80]. The disruption of TADs has been reported to rewire chromatin structure interactions between promoters and enhancers, which leads to human limb malformations [81].

For validation and complement analysis on genomic 3C-based profiling techniques, the recent molecular imaging for noninvasive detection, such as quantitative 3D telomere fluorescence in situ hybridization (FISH) analysis and 3D super-resolution imaging (3D-SIM), are also a promising direction [82,83].

## Figures and Tables

**Table 1:** Typical tools for Hi-C data analysis.

Tool	Sequencing Reads Aligner	Feature	Programing Language
HiCapp [44]	Bowtie2	Correct copy number bias	Shell
hiclib [45]	Bowtie2	Iterative	Python
HiCExplorer [46]	Bwa, Bowtie2, Hisat2	Check inter chromosomal fraction of reads	Python
HiC-Pro [47]	Bowtie2	Trimming of reads	Python, R
TADbit [48]	GEM	Iterative	Python
HiCUP [49]	Bowtie, Bowtie2	Pre-truncation	Perl, R
HiC-Box [50]	Bowtie2	Correct contact maps for systematic biases	Python
HiCdat [51]	Subread	Analyze larger structural features	C++, R
HIPPIE [52]	BWA	Extract enhancer-target gene relationships	Python, Perl, R
HiC-inspector [53]	Bowtie	Focus on mapping and filtering	Perl, R
